# Remineralisation capability of silver diamine fluoride in artificial enamel lesions on smooth surfaces using quantitative light-induced fluorescence measurements in-vitro

**DOI:** 10.1038/s41598-022-12498-6

**Published:** 2022-05-19

**Authors:** J. Heukamp, H. Korbmacher-Steiner, S. Schmidt, C. M. Neumann, P. Bottenberg, A. Jablonski-Momeni

**Affiliations:** 1 Department of Orthodontics, Dental School, Philipps University of Marburg, Georg-Voigt-Str. 3, 35039 Marburg, Germany; 2 School for Oral Health Sciences, Free University of Brussels (ULB), Brussels, Belgium

**Keywords:** Health care, Dentistry

## Abstract

Enamel demineralisation can develop on smooth surfaces as an undesirable side effect during orthodontic treatment with fixed appliances. This study aimed to evaluate the ability of 38% silver diamine fluoride in remineralisation (as estimated by fluorescence gain) of artificial initial lesions in smooth surfaces of human enamel. The smooth surfaces of 50 human tooth samples were artificially demineralised and 45 samples were allocated randomly into three groups receiving a single treatment with a varnish: group I: Riva Star (silver diamine fluoride, SDF), group II: Bifluorid 12 (NaF, CaF_2_), and group III: Cervitec F (CHX, CPC, NH_4_F). Five samples were assigned as a negative control group without treatment. All samples were exposed to pH-cycling for 28 days. Fluorescence behavior was measured using Quantitative light-induced fluorescence before and after demineralisation and up to four weeks on a weekly basis. Analysis of variance (ANOVA) with Tukey–Kramer post-hoc tests and repeated measures ANOVA were used for statistical evaluation (α = 0.05). After demineralisation, all samples showed mean ΔF of − 16.22% ± 4.35, without significance differences between the fluorescence behaviour of the samples (*p* = 0.251). After 28 days group comparison showed a statistically significant difference (*p* = 0.034) for ΔF values: the lowest fluorescence values were found in group I (SDF, mean ΔF − 16.47 ± 6.08) with a significant difference compared to group III (Cervitec F, mean ΔF − 11.71 ± 4.83). In group II (Bifluorid 12) mean ΔF value was − 15.55 ± 2.15) without statistically significant differences to groups I and III. The fluorescence behaviour of SDF varnish on smooth surfaces with artificial initial enamel lesions was significantly lower compared to Cervitec F varnish after short time use.

## Introduction

Dental caries is the most prevalent chronic disease in the world^1^. The symptoms and consequences of caries, such as pain, chewing problems and aesthetic limitations due to discolouration, damage and loss of teeth, have a major impact on quality of life^2^. Before caries appears in its irreversible form, it passes through the stage of initial caries during de- and remineralisation cycles^3^. At that stage its progression can be arrested or reversed by application of fluorides^4^. Applying fluoride with different formulations was shown to prevent dental caries effectively^5,6^. In low-risk patients, the daily use of fluoridated toothpaste is sufficient. In patients presenting an elevated risk of carious lesion progression, application forms with a higher concentration of fluoride or containing fluoride in combination with other active compounds may be advisable.

The use of a 38% solution silver diamine fluoride (SDF) has been shown to be an effective method for arresting advanced cavitated carious lesions^7^. Due to its low acquisition costs and its synergistic caries-preventing effects, it has so far been used mainly in developing and emerging countries^8^. The disadvantage of a black staining of tooth surfaces precludes its application in patients with aesthetic demands such as adolescents. However, recent changes in formulation by its combination with potassium iodide, has reduced the stain development at least when applied to enamel^9^. Silver ions have a positive effect on the microhardness of carious surfaces^10^ and increase mineral density in carious lesions significantly compared with fluoridated toothpaste alone, especially in lesion’s superficial layers^11^.

Orthodontic treatment with fixed appliances is an integral part of modern orthodontics, but it has also been associated with adverse effects like development of initial carious lesions. Such lesions might progress without appropriate preventive care^12^ and have a negative effect on the esthetic and functional outcome of orthodontic treatment^13^. It was reported that the incidence of carious lesions formed during orthodontic treatment in patients amounted to 45.8%^14^. Initial lesions can develop within the first four weeks of fixed appliance treatment^15^. Based on the existing trials, interventions for post-orthodontic WSLs, mainly fluoride varnish, seem to be effective, but further research is needed to elucidate their clinical relevance^16^.

While the effectiveness of SDF in arresting dentine lesions and root caries has been already shown^7,17^, only few studies exist on the use of SDF on initial enamel lesions on smooth surfaces. Therefore, the aim of the present study was to evaluate the remineralisation potential of the KI-modified SDF on artificial initial enamel lesions on smooth surfaces compared to the use of two conventional fluoride-containing dental varnishes.

## Results

In total, 50 samples were included in the study: n = 15 in each treatment group, n = 5 in the negative control group.

After demineralisation, all samples showed a distinct fluorescence loss with a mean ΔF of − 16.22% ± 4.35 and a mean ΔFmax of − 33.40% ± 6.82, respectively. Group comparison revealed no significant differences between the fluorescence behaviour of samples (*p* = 0.251 for ΔF and *p* = 0.276 for ΔFmax), indicating a comparable fluorescence behaviour in each group after demineralisation.

The results of the QLF-measurements (average percentage of fluorescence loss relative to the fluorescence of sound tissue, ΔF, %) and maximum lesion depth (ΔFmax, %) are summarized in Tables 1 and 2. QLF measurements at baseline (prior to demineralisation) showed ΔF and ΔFmax values of 0 for each specimen, indicating a tooth surface without demineralisation when compared to sound surrounding tissue.

**Table 1:** Results of the QLF measurements in different groups: ΔF (fluorescence loss [%]) shows avarage fluorescence loss in a lesion and is related to the loss of mineral content and to lesion depth (SD: standard deviation).

Time	Group	Minimum	Maximum	Mean	SD	Group comparison
ΔF T-demin	I: SDF	− 21.3	− 8.1	− 16.65	4.13	*p* = 0.251
	II: Bifluorid 12	− 22.7	− 7.2	− 15.38	4.70	
	III: Cervitec F	− 23.8	− 10.0	− 17.56	4.16	
	Negative control	− 19.1	− 10.1	− 13.46	3.89	
ΔF T07	I: SDF	− 21.8	− 8.4	− 14.16	3.94	*p* = 0.075
	II: Bifluorid 12	− 30.5	− 10.5	− 17.88	4.89	
	III: Cervitec F	− 24.1	− 8.1	− 15.88	4.13	
ΔF T14	I: SDF	− 23.1	− 8.6	− 15.53	4.42	*p* = 0.060
	II: Bifluorid 12	− 30.9	− 12.6	− 19.40	5.19	
	III: Cervitec F	− 21.9	− 7.4	− 15.97	4.56	
ΔF T21	I: SDF	− 31.3	− 8.6	− 16.17	6.81	*p* = 0.131
	II: Bifluorid 12	− 27.1	− 12.1	− 18.81	4.12	
	III: Cervitec F	− 22.0	− 8.9	− 15.08	3.81	
ΔF T28	I: SDF	− 33.0	− 9.9	− 16.47^a^	6.08	*p* = 0.034*
	II: Bifluorid 12	− 20.0	− 12.4	− 15.55^a,b^	2.15	
	III: Cervitec F	− 22.9	− 6.3	− 11.71^b^	4.83	
	Negative control	− 20.2	− 12.6	− 15.40^a,b^	3.20	

*Different superscript letters indicate significant differences between the groups.

**Table 2:** Results of the QLF measurements in different groups: ΔF max (fluorescence loss [%]) shows the highest value of ΔF measured within the region of interest and is an indication for the maximum lesion depth (SD: standard deviation).

Time	Group	Minimum	Maximum	Mean	SD	Group comparison
ΔFmax T-demin	I: SDF	− 45.8	− 20.3	− 33.97	6.75	*p* = 0.276
	II: Bifluorid 12	− 41.4	− 13.2	− 30.69	7.94	
	III: Cervitec F	− 50.3	− 26.4	− 35.51	6.07	
	Negative control	− 40.1	− 30.5	− 33.56	3.89	
ΔFmax T07	I: SDF	− 44.8	− 22.0	− 33.17	6.55	*p* = 0.152
	II: Bifluorid 12	− 49.6	− 27.5	− 37.66	6.87	
	III: Cervitec F	− 43.5	− 20.7	− 34.32	5.86	
ΔFmax T14	I: SDF	− 50.0	− 28.6	− 37.96^a,b^	8.07	*p* = 0.021*
	II: Bifluorid 12	− 50.0	− 30.2	− 40.41^a^	6.20	
	III: Cervitec F	− 40.0	− 19.3	− 33.01^b^	6.91	
ΔFmax T21	I: SDF	− 59.5	− 21.2	− 39.23	11.60	*p* = 0.090
	II: Bifluorid 12	− 61.8	− 28.6	− 39.89	8.32	
	III: Cervitec F	− 41.0	− 19.4	− 33.18	6.14	
ΔFmax T28	I: SDF	− 52.4	− 26.8	− 38.14^a^	8.71	*p* < 0.001*
	II: Bifluorid 12	− 50.9	− 26.2	− 34.53^a^	6.37	
	III: Cervitec F	− 46.8	− 9.80	− 24.24^b^	9.98	
	Negative control	− 45.2	− 30.6	− 34.98^a,b^	6.00	

*Different superscript letters indicate significant differences between the groups.

After application of varnish and pH-cycling, repeated measures ANOVA showed that neither ΔF nor ΔFmax values changed significantly between measurements after demineralisation and the values at the end point of the study at T28 within group I (*p* = 0.713 and 1.00, respectively). The same results were observed in group II, where the fluorescence values did not change between T-demin and T28 (p values for and ΔF and ΔFmax = 1.00). In group III, significant fluorescence gain was observed between T-demin and T28 for ΔF (*p* = 0.012) and ΔFmax (*p* = 0.011). Data of the control group showed lower fluorescence values for ΔF and ΔFmax at T28, compared to the values after demineralisation. In all groups, comparison of fluorescence values at baseline showed significant differences to the values after demineralisation and at each follow-up measurement (*p* < 0.0001).

The group comparison (ANOVA with Tukey–Kramer test) showed significantly lower fluorescence values in the SDF-group compared to Cervitec F after 28 days (*p* = 0.034 for ΔF and *p* < 0.001 for ΔFmax).

In group I a yellow discoloration of the enamel surfaces in all samples was obvious 24 h after application.

**Figure 1:**
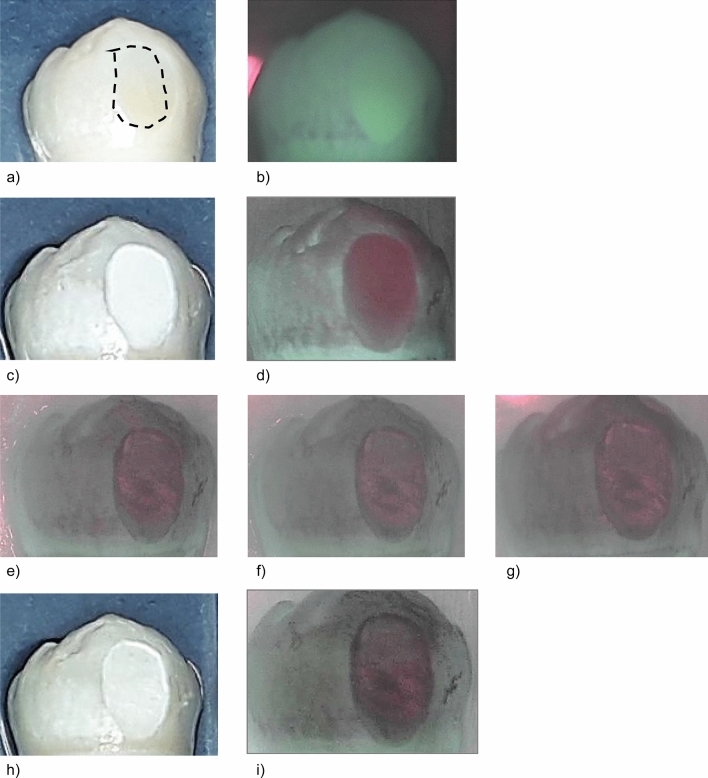
Standard image and QLF image of a representative sample in group I (SDF, sample #05) at different investigation times. The dashed line indicates the ground surface where the QLF measurements were performed. (**a**) Prior to demineralization. (**b**) Corresponding QLF-image: ΔF = 0.0%; ΔFmax = 0.0%. (**c**) After demineralisation (T-demin). (**d**) Corresponding QLF-image (T-demin): ΔF = − 17.0%; ΔFmax = − 34.1%. (**e**) QLF image T07: ΔF = − 12.2%; ΔFmax = − 28.6%. (**f**) QLF image T14: ΔF = − 12.8%; ΔFmax = − 30.2%. (**g**) QLF image T21: ΔF = − 11.5%; ΔFmax = − 33.3%. (**h**) 28 days after demineralisation (T28). (**i**) Corresponding QLF-image (T28): ΔF = − 14.5%; ΔFmax = − 30.2%.

**Figure 2:**
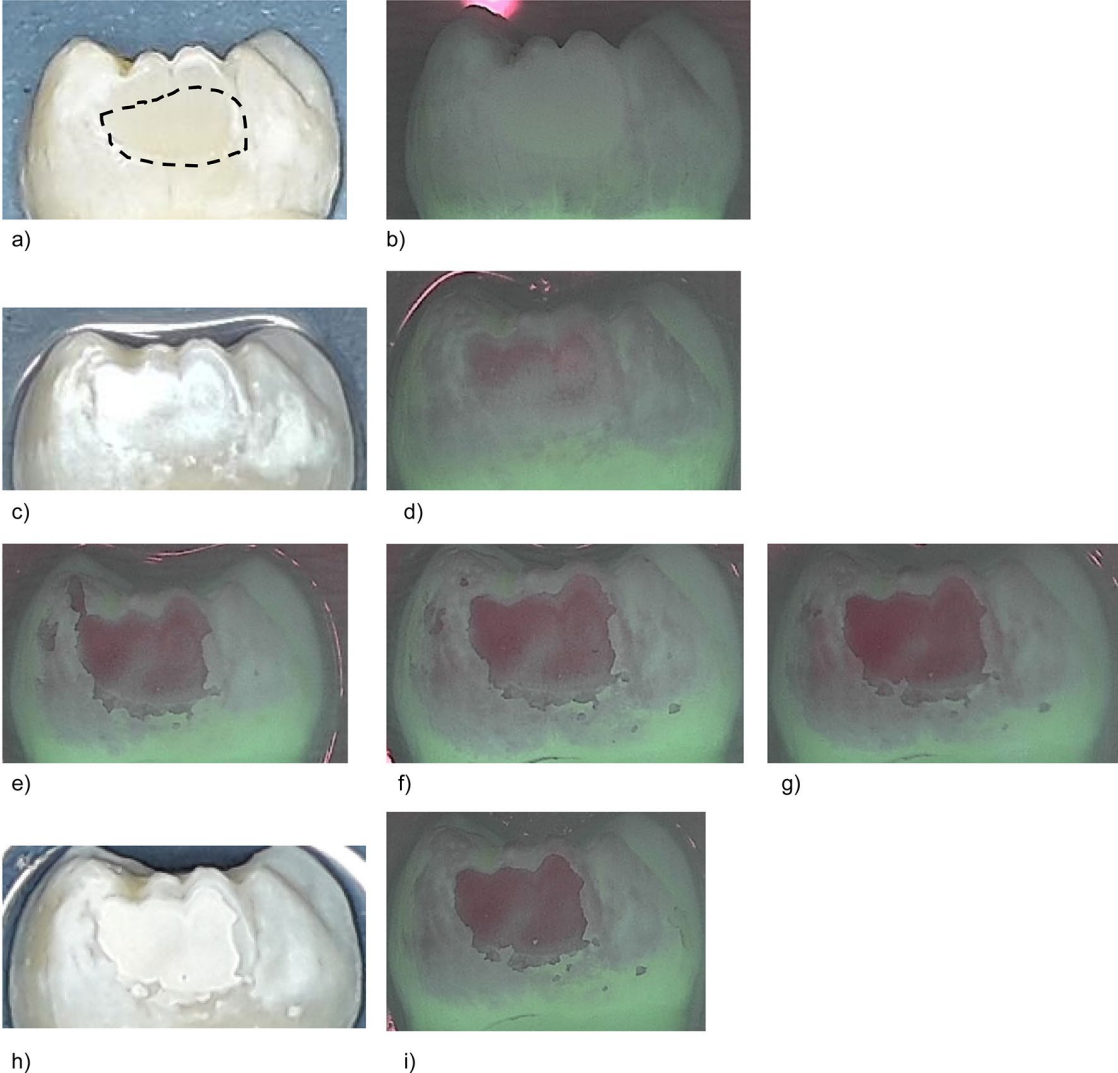
Standard image and QLF image of a representative sample in group II (Bifluorid 12, sample #12) at different investigation times. The dashed line indicates the ground surface where the QLF measurements were performed. (**a**) Prior to demineralisation. (**b**) Corresponding QLF-image: ΔF = 0.0%; ΔFmax = 0.0%. (**c**) After demineralisation (T-demin). (**d**) Corresponding QLF-image (T-demin): ΔF = − 11.3%; ΔFmax = − 20.9%. (**e**) QLF image T07: ΔF = − 17.7%; ΔFmax = − 36.7%. (**f**) QLF image T14: ΔF = − 17.1%; ΔFmax = − 36.2%. (**g**) QLF image T21: ΔF = − 13.3%; ΔFmax = − 30.1%. (**h**) 28 days after demineralisation (T28). (2**i**) Corresponding QLF-image (T28): ΔF = − 15.7%; ΔFmax = − 34.2%.

**Figure 3:**
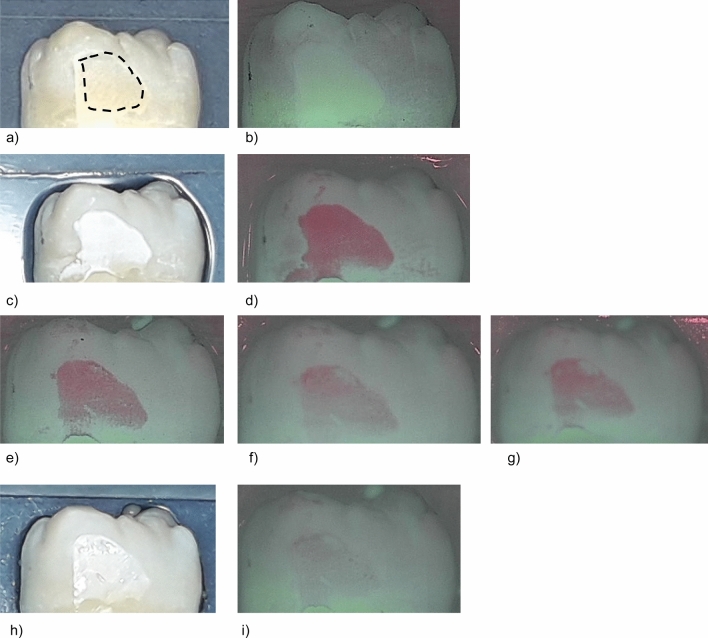
Standard image and QLF image of a representative sample in group III (Cervitec F, sample #09) at different investigation times. The dashed line indicates the ground surface where the QLF measurements were performed. (3**a**) Prior to demineralization. (**b**) Corresponding QLF-image: ΔF = 0.0%; ΔFmax = 0.0%. (**c**) After demineralisation (T-demin). (**d**) Corresponding QLF-image (T-demin): ΔF = ΔF = − 16.5%; ΔFmax = − 30.5%. (**e**) QLF image T07: ΔF = − 14.7%; ΔFmax = − 27.1%. (**f**) QLF image T14: ΔF = − 8.9%; ΔFmax = − 19.8%. (**g**) QLF image T21: ΔF = − 9.8%; ΔFmax = − 19.4%. (**h**) 28 days after demineralisation (T28). (**i**) Corresponding QLF-image (T28): ΔF = − 6.3%; ΔFmax = − 9.8%.

**Figure 4:**
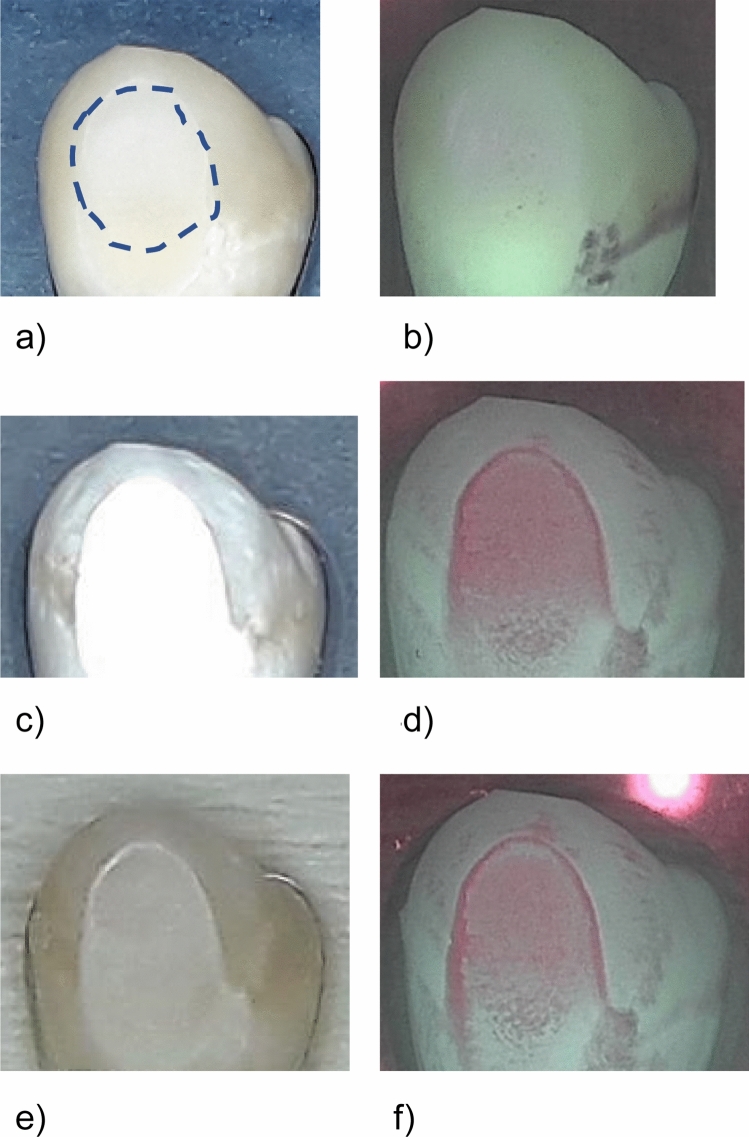
Standard image and QLF image of a representative sample in the negative control group (no treatment, sample #09) at different investigation times. The dashed line indicates the ground surface where the QLF measurements were performed. (**a**) Prior to demineralisation. (**b**) Corresponding QLF-image: ΔF = 0.0%; ΔFmax = 0.0%. (**c**) After demineralisation (T-demin). (**d**) Corresponding QLF-image (T-demin): ΔF = − 10.4%; ΔFmax = − 33.7%. (**e**) 28 days after demineralisation (T28). (**f**) Corresponding QLF-image (T28): ΔF = − 12.6%; ΔFmax = − 33.5%.

In Figs. 1, 2, 3 and 4, representative images of each treatment group with the corresponding fluorescence values are presented. The measurements were performed in the center of sample with the ground surface.

## Discussion

Caries progression is initiated by an imbalance of the remineralisation–demineralisation equilibrium favoring demineralisation^18^. Initial caries can occur as side effect of fixed orthodontic treatment and prevention is important during but also after the treatment to prevent initial lesions from progression into dentin. In the present study, the ability of Silver diamine fluoride (SDF) was evaluated to remineralise artificial enamel lesions.

Due to its antimicrobial and caries inhibitory properties, SDF promises to be a good and affordable option to treat such reversible initial caries lesions.

In this study, extracted human permanent teeth were used. This offered the advantage that the chemical structure of the tooth samples corresponded to that in patients. The cleaning and flattening of the tooth surface eliminated external influences as far as possible and made the teeth more comparable with each other. The caries model used in this study aims to represent the demineralisation/remineralisation dynamics of the caries process as accurately as possible. The procedure of demineralising the enamel surface was based on an already described protocol^19^ and the pH-cycling model was modified according to available procedures and equipment. This may show a limitation of the present study since the pH-cycling model is not fully comparable to already existing studies.

In contrast to the in vivo situation, no cariogenic bacteria and biofilm were used in the demineralisation solution. This makes it difficult to transfer the results from in vitro to in vivo, but makes the present study more comparable with other in vitro studies that also excluded the biological component^20–23^.

In the present study, the remineralisation performance of the fluoride varnishes investigated was determined using quantitative light-induced fluorescence. QLF is a versatile and non-destructive method established in several studies^19,24–26^. The accuracy is comparable to that of transverse microradiography, which is considered the gold standard^27^, but needs sectioning of the samples.

Using the latest version of QLF an additional control group is not necessary, since the control is carried out via the measurement of the surfaces after demineralisation and before the application of the varnishes and start of pH-cycling. This type of internal control also serves the purpose of the present study, which is to compare the different fluoride varnishes. This approach is also established in the literature for comparative in vitro studies using QLF ^19,26,28–30^. Though, an untreated group of samples (n = 5) was included as quality control in the present study and the fluorescence changes were compared between T-demin and T28. Since the fluorescence behaviour at T-demin was comparable for all samples in each group, the measurements were set as the reference values for comparison of the remineralisation behaviour of the different varnishes in a defined study period. The fluorescence behaviour of the group without treatment was comparable to the results of another study where no fluorescence gain was observed after 4 weeks of storage in a remineralisation solution^19^.

It should be noted that with the new QLF device used in this study, the reference of a surface (sound surface value) is determined automatically by the integrated software. This is different from the former QLF device where the sound reference surface was determined manually^19^. To ensure quality and correctness of data assessment, the QLF measurements were also performed prior to demineralisation on the sound surfaces showing typically fluorescence values 0 for each specimen, indicating a sound tooth surface. Hence, the fluorescence values measured at T-demin reveal the accurate fluorescence behaviour.

The fluorescence values show different trends for each group. While group I appears to follow a slow demineralising trend, the mineralisation of groups II and III drops more sharply than that of Group I up to T14, but recovers thereafter and exceeds mineralisation of Group I at T28. This pattern can be observed in both ΔF and ΔFmax values.

Especially in the last few years, many studies using different methods have been conducted on SDF, alone or in comparison with other products. Erdwey et al.^31^ were able to demonstrate through transverse microradiography that a commercially available NaF varnish (Bifluorid 12) has a better remineralisation behaviour than SDF, amine fluoride and acidulated phosphate fluoride (APF) after 28 days of pH-cycling. Also using TMR, Wierichs et al.^32^ demonstrated that SDF could achieve remineralisation after 14 days of pH-cycling, but NaF could not. In other studies tooth samples treated with SDF and NaF were measured by Vickers hardness^23^. The NaF-treated tooth sample had a higher hardness than the SDF-treated one after 7 days of pH-cycling. Other methods of measurement seem to attribute SDF a better remineralisation performance than NaF: De Melo Santos et al.^20^ used polarised light microscopy (PLM) after 14 days of pH-cycling, Yu et al.^22^ utilised energy dispersive radiospectroscopy after 21 days of pH-cycling and Mohammadi and Far^21^ used surface micro hardness after 7 days of pH-cycling. Lastly Thomas et al.^33^ using swept-source optical coherence tomography described better remineralisation results for SDF after 14 days of pH-cycling compared to NaF.

One disadvantage of SDF which was observed in the present study was the yellow discoloration of the samples appearing after 24 h. Even after pH-cycling and brushing the teeth, the discoloration remained. This esthetic shortcoming makes it difficult to recommend SDF for use in surfaces in the visible area such as anterior teeth. While a yellow discoloration is not described for SDF in literature yet, it could be caused by the precipital reaction of SDF with potassium iodide (KI). KI is part of the Riva Star System to minimize gray staining of the teeth^34^. The precipitate is mentioned in literature^35^ and might consist of silver iodide^36^. In our study, the precipitate was also found in the remineralising solution, where the teeth treated with SDF were stored, presenting itself as cloud-like structures. Looking at the fluorescence values, it can be assumed that this discoloration did not affect QLF measurements of specimen treated with SDF, otherwise significant differences would have been found between mean ΔF values already at an early stage of the study.

## Conclusion and clinical relevance

The presented study is the first study to evaluate the fluorescence behaviour of artificial initial enamel lesions after a single treatment with SDF on a weekly basis for four weeks. This duration takes into account that initial lesions in caries risk patients such as those with fixed orthodontic appliances can develop on smooth surfaces within four weeks. Hence appropriate preventive care is already required from the beginning. The presented data suggest that SDF can prevent progression of demineralisation which is clinically relevant in patients who are temporarily restricted in their individual oral hygiene or where mechanical obstacles such as fixed orthodontic appliances are present. However, the fluorescence behaviour of SDF varnish on smooth surfaces was lower compared to Cervitec F and Bifluorid 12 varnishes after short time use.

## Methods

### Sample selection and preparation

The use of extracted human teeth was approved by the Ethics Committee of the Medical Faculty of the Philipps-University of Marburg, Germany (Ref. No.: 132/19). Prior to extraction informed consent was obtained from each patient for the use of the teeth for study purposes. The research did not involve human participants.

A sample size calculation was performed with MedCalc for Windows, version 19.6 (MedCalc Software, Ostend, Belgium, www.medcalc.org) based on (unpublished) pilot data. A number of 13 samples was calculated for each treatment group (Power 0.90, α = 0.05), assuming a mean difference of 4 and a standard deviation of 3 in each group. A drop-out number of two samples was added in each group. The teeth were stored in a 0.001% sodium azide solution after extraction for disinfection and were cleaned afterwards. The crown was separated from the root and then cut along the mesiodistal direction using a water-cooled 200 μm diamond band saw (EXAKT 300/310 CP, EXAKT Advanced Technologies GmbH, Norderstedt, Germany). Subsequently the surfaces were ground with a micro-grinding device (EXAKT 400CS, EXAKT Advanced Technologies GmbH, Norderstedt, Germany) with a grit size of 1000 μm. By removing approx. 50 μm, a plane smooth surface located in the enamel was thus created in each tooth sample.

45 tooth samples were randomly divided into 3 groups with n = 15 samples each: Group I: Riva Star (SDI limited, Baywater, Australia) (38% silver fluoride (AgF), amounting to 5% fluoride and an additional potassium iodide solution); group II: Bifluorid 12, containing 6% NaF and 6% CaF_2_ (VOCO GmbH, Cuxhaven, Germany) and group III: Cervitec F, containing 0.3% chlorhexidine, 0.5% cetylpyridiniumchloride and 1400 ppm F^-^ (as NH_4_F) (Ivoclar Vivadent AG, Schaan, Liechtenstein). In addition, 5 samples were allocated to a negative control group, without receiving any varnish treatment after demineralisation.

The samples were placed on microscope slide (Plexiglas slide 25 mm × 75 mm × 2 mm, EXAKT Advanced Technologies GmbH, Norderstedt, Germany) so that the ground enamel surfaces pointed upwards horizontally.

### Demineralisation of the samples

The process of demineralisation was already described in detail by Jablonski-Momeni et al.^19^. In brief, the samples were demineralised in lactic acid (pH 4.6) buffered by a methylcellulose lactic acid gel for 14 days according to the literature^37^. The mean depth of the demineralised area amounted to 97.32 μm, analysed in representative histological sections in a preliminary study^19^.

### Application of the varnishes

The tooth samples were air-dried and each varnish was applied to the samples according to the corresponding manufacturer's instructions. In case of SDF additional application of potassium iodide (KI) was performed. Afterwards, any liquid excess of the varnishes was carefully patted dry with a cotton pellet.

### pH-cycling

The pH-cycling in the presented study was performed according to the following protocol: The remineralisation solution was prepared by mixing 1.635 g KH_2_PO4 (2.403 mM), 1.700 g Na_2_HPO_4_ (2.395 mM), 6.335 g KCL (16.993 mM), 2.920 g NaCl (9.993 mM) and 0.830 g CaCl_2_·2 H_2_O (1.129 mM) in 5 L distilled water^19^ (all chemicals: Merck, Darmstadt, Germany). The pH was adjusted to 6.9 and was measured daily. For demineralisation a 0.1 M lactic acid solution was used with a pH of 4.6 (Lactic Acid, Fluka Analytical, Honeywell International Inc., Seelze, Germany).

Toothpaste slurries were used to simulate tooth brushing. These were prepared as a water/toothpaste slurry from 50 ml distilled water and a 20 cm strand (~ 16 g) of commercial toothpaste containing 1450 ppm fluoride (as NaF) (Colgate Komplett Extra Frisch, CP GABA GmbH, Hamburg, Germany) corresponding to a ratio of approximately 3:1. The samples were gently brushed with a commercial toothbrush (Colgate Extra Clean Mittel, CP GABA GmbH, Hamburg, Germany). Per group a separate toothbrush was used to avoid carry-over.

The cycle was performed manually twice a day. In the first step the samples were removed from the remineralising solution and submerged in the demineralisation solution for 2 min. Afterwards they were submerged in the slurry and brushed with a manual toothbrush for 2 min. Thereafter, the samples were returned to the remineralising solution. After submersion in each of the solutions, the samples were rinsed with distilled water to prevent carry-over. The de- and remineralisation solutions were replaced after 7 days. Between measurements the samples were stored in the incubator at a constant temperature of 37 °C.

### Quantitative light-induced fluorescence (QLF)

Noninvasive measurements were performed as the reference standard for the quantification of de- and remineralisation using the Quantitative light induced fluorescence method. The QLF analysis software C4 QLF Research Suite 1.08 (Inspector Research Systems BV, Amsterdam, Netherlands) was used to quantify the fluorescence behavior of the enamel surfaces which is directly proportional to the mineral content. Using the new QLF device, the fluorescence value of a sound surface is determined automatically by the software in contrast to the former QLF device where the sound reference surface was to be determined manually^19^.

Measurements in the treatment groups were performed after demineralisation (T-demin) and after 7 days (T-07), 14 days (T-14), 21 days (T-21) and 28 days (T-28) of application of the fluoride varnishes. Measurements in the control group were performed at T-demin and T28. The ground parts of the enamel were defined in each sample and this area was recalled on the following images (Figs. 1, 2 , 3 and 4). Average percentage of fluorescence loss relative to the fluorescence of sound tissue (ΔF, %) and maximum lesion depth (ΔFmax, %) was calculated by the software und the values were used for further analyses. Negative ΔF values indicate an increase of lesion depth or mineral loss.

By way of internal quality control, the QLF measurements were also performed prior to demineralisation on the sound surfaces showing typically ΔF values 0 for each specimen, indicating a sound tooth surface.

### Statistical evaluation

Statistical analyses were performed using MedCalc for Windows, version 19.6 (MedCalc Software, Ostend, Belgium, www.medcalc.org). Data were tested using the Shapiro–Wilk’s test to check for normality (*p* > 0.05). Analysis of variance (ANOVA) with Tukey–Kramer post-hoc tests and repeated measures ANOVA were used for further analyses. The significance level was set at α = 0.05.

### Ethical approval

The use of the extracted teeth was subject to an ethics vote by the ethics committee of the Philipps University of Marburg (Ref. No.: 132/19). Informed consent was obtained from each patient for the use of the teeth for study purposes prior to extraction. The research did not involve human participants. All methods were carried out in accordance with relevant guidelines and regulations. The attribution guidelines for the software MedCalc were followed.

## Data Availability

The dataset analysed during the current study is available from the corresponding author on reasonable request.

## References

[1] Kassebaum NJ (2015). Global burden of untreated caries: a systematic review and metaregression. J. Dent. Res..

[2] Petersen PE, Bourgeois D, Ogawa H, Estupinan-Day S, Ndiaye C (2005). The global burden of oral diseases and risks to oral health. Bull. World Health Organ..

[3] Machiulskiene V (2020). Terminology of dental caries and dental caries management: consensus report of a workshop organized by ORCA and cariology research group of IADR. Caries Res..

[4] Zampetti P, Scribante A (2020). Historical and bibliometric notes on the use of fluoride in caries prevention. Eur. Arch. Paediatr. Dent..

[5] Marinho VC, Higgins JP, Logan S, Sheiham A (2003). Topical fluoride (toothpastes, mouthrinses, gels or varnishes) for preventing dental caries in children and adolescents. Cochrane Database Syst. Rev..

[6] Marinho VC, Worthington HV, Walsh T, Clarkson JE (2013). Fluoride varnishes for preventing dental caries in children and adolescents. Cochrane Database Syst. Rev..

[7] Urquhart O (2019). Nonrestorative treatments for caries: systematic review and network meta-analysis. J. Dent. Res..

[8] Mattos-Silveira J (2014). New proposal of silver diamine fluoride use in arresting approximal caries: study protocol for a randomized controlled trial. Trials.

[9] Haiat A, Hien CN, Lakshman PS, Kausar SF (2021). The effect of the combined use of silver diamine fluoride and potassium iodide in disrupting the plaque biofilm and alleviating tooth disoloration: a systemic review. PLoS One.

[10] Firouzmandi M, Shafiei F, Jowkar Z, Nazemi F (2019). Effect of silver diamine fluoride and proanthocyanidin on mechanical properties of caries-affected dentin. Eur. J. Dent..

[11] Punyanirun K, Yospiboonwong T, Kunapinun T, Thanyasrisung P, Trairatvorakul C (2018). Silver diamine fluoride remineralized artificial incipient caries in permanent teeth after bacterial pH-cycling in-vitro. J. Dent..

[12] Enaia M, Bock N, Ruf S (2011). White-spot lesions during multibracket appliance treatment: a challenge for clinical excellence. Am. J. Orthod. Dentofacial Orthop..

[13] Gorelick L, Geiger AM, Gwinnett AJ (1982). Incidence of white spot formation after bonding and banding. Am. J. Orthod. Dentofacial Orthop..

[14] Sundararaj D, Venkatachalapathy S, Tandon A, Pereira A (2015). Critical evaluation of incidence and prevalence of white spot lesions during fixed orthodontic appliance treatment: a meta-analysis. J. Int. Soc. Prevent. Commun. Dent..

[15] Ogaard B, Rolla J, Arends J (1998). Orthodontic appliances and enamel demineralization. Part 1. Lesion development. Am. J. Orthod. Dentofacial Orthop..

[16] Höchli D, Hersberger-Zurfluh M, Papageorgiou SN, Eliades T (2017). Interventions for orthodontically induced white spot lesions: a systematic review and meta-analysis. Eur. J. Orthod..

[17] Oliveira BH, Cunha-Cruz J, Rajendra A, Niederman R (2018). Controlling caries in exposed root surfaces with silver diamine fluoride: a systematic review with meta-analysis. J. Am. Dent. Assoc..

[18] Featherstone JD (2004). The continuum of dental caries—evidence for a dynamic disease process. J. Dent. Res..

[19] Jablonski-Momeni A (2020). Impact of self-assembling peptides in remineralisation of artificial early enamel lesions adjacent to orthodontic brackets. Sci. Rep..

[20] De Melo Santos L, dos Reis JIL, de Medeiros MP, Ramos SM, de Araúyo JM (2009). In vitro evaluation of fluoride products in the developement of carious lesions in deciduous teeth. Braz. Oral Res..

[21] Mohammadi N, Far MHF (2018). Effect of fluoridated varnish and silver diamine fluoride on enamel demineralization resistance in primary dentition. J. Indian Soc. Pedod. Prev. Dent..

[22] Yu OY (2018). Remineralisation of enamel with silver diamine fluoride and sodium fluoride. Dent Mater..

[23] Akyildiz M, Sönmez S (2019). Comparison of remineralising potential of nano silver fluoride, silver diamine fluoride and sodium fluoride varnish on artificial caries: an in vitro study. Oral Health Prev. Dent..

[24] Boersma JG, van der Veen MH, Lagerweij MD, Bokhout B, Prahl-Andersen B (2005). Caries prevalence measured with QLF after treatment with fixed orthodontic appliances: influencing factors. Caries Res..

[25] Gokce G, Savas S, Kucukyilmaz E, Veli I (2017). Effects of toothpastes on white spot lesions around orthodontic brackets using quantitative light-induced fluorescence (QLF): an in vitro study. J. Orofac. Orthop..

[26] Jablonski-Momeni A, Sambale J, Gaerttner L, Nothelfer R, Korbmacher-Steiner H (2021). Use of bioluminescence measurements for detection of artificial demineralization adjacent to orthodontic brackets. J. Orofac. Orthop..

[27] Pretty IA, Smith PW, Edgar WM, Higham SM (2003). Detection of in vitro demineralization adjacent to restorations using quantitative light induced fluorescence (QLF). Dent. Mater..

[28] Topsakal KG, Amuk NG (2019). Effects of different remineralisation agents and adhesives around orthodontic brackets: is there a relationship between remineralisation and shear bond strength. Oral Health Prev. Dent..

[29] Ajaj MK, Al-Khateeb SN, Al-Batayneh OB (2020). Effect of different acid etchants on the remineralization process of white-spot lesions: an in vitro study. Am. J. Dent..

[30] Dhanya K, Chandra P, Anandakrishna L, Karuveettil V (2021). A comparison of NovaMin™ and casein phosphopeptide-amorphous calcium phosphate fluoride on enamel remineralization: an in vitro study using scanning electron microscope and DIAGNOdent ^R^. Contemp. Clin. Dent..

[31] Erdwey D, Meyer-Lueckel H, Esteves-Oliveira M, Apel C, Wierichs RJ (2021). Demineralization inhibitory effects of highly concentrated fluoride dentifrice and fluoride gels/solutions on sound dentin and artificial dentin caries lesions in vitro. Caries Res..

[32] Wierichs RJ, Stausberg S, Lausch J, Meyer-Lueckel H, Esteves-Oliveira M (2018). Caries preventive effect of NaF, NaF plus TCP, NaF plus CPP-ACP, and SDF varnishes on sound dentin and artificial dentin caries in vitro. Caries Res..

[33] Thomas CS, Sharma DS, Sheet D, Mukhopadhyay A, Sharma S (2021). Cross-sectional visual comparison of remineralization efficacy of various agents on early smooth surface caries of primary teeth with swept source optical coherence tomography. J. Oral. Biol. Craniofac. Res..

[34] Abdullah N (2020). The antibacterial efficacy of silver diamine fluoride (SDF) is not modulated by potassium iodide (KI) supplements: a study on in-situ plaque biofilms using viability real-time PCR with propidium monoazide. PLoS One.

[35] Hamama HH, Yiu CK, Burrow MF (2015). Effect of silver diamine fluoride and potassium iodide on residual bacteria in dentinal tubules. Aust. Dent. J..

[36] Jander, G. & Blasius, E. Elemente der 7. Hauptgruppe. in *Anorganische Chemie I*—*Einführung & Qualitative Analyse 17. Aktualisierte Auflage* (ed. Schweda, E.) 171–200 (Hitzel Verlag Stuttgart, 2012).

[37] ten Cate JM (1996). Preparation and measurement of artificial enamel lesions, a four-laboratory ring test. Caries Res..

